# Growth hormone deficiency in three siblings homozygous for a rare *GH1* haplotype

**DOI:** 10.3389/fendo.2025.1704842

**Published:** 2026-01-05

**Authors:** Ana Cláudia Ribeiro, Omneya Magdy Omar, Ebtesam Abdalla, Manuel Carlos Lemos

**Affiliations:** 1 RISE-Health, Faculty of Health Sciences, University of Beira Interior, Covilhã, Portugal; 2 Department of Pediatrics, Faculty of Medicine, Alexandria University, Alexandria, Egypt; 3 Department of Human Genetics, Medical Research Institute, Alexandria University, Alexandria, Egypt

**Keywords:** growth hormone deficiency, GH deficiency, IGHD, GH1, haplotype, promoter polymorphism, transcriptional regulation

## Abstract

**Introduction:**

Growth Hormone (GH), secreted by the anterior pituitary gland, is a key regulator of postnatal growth. Mutations in the *GH1* gene can lead to isolated GH deficiency (IGHD), a rare disorder characterized by growth failure and severe short stature. The aim of this study was to identify the genetic basis of IGHD in three siblings born to consanguineous parents.

**Methods:**

Three siblings were diagnosed with short stature due to GH deficiency (stimulated GH peak levels between 0.07 and 0.77 µg/L). To identify their genetic cause, whole-exome sequencing (WES), multiplex ligation-dependent probe amplification (MLPA), and targeted *GH1* sequencing was performed.

**Results:**

A shared homozygous *GH1* haplotype comprising nine single nucleotide polymorphisms (SNPs), spanning the promoter, coding, and 3’ flanking regions, was revealed. The parents were heterozygous carriers of this haplotype. This rare SNP combination (with less than 1% population frequency) overlaps with transcriptional regulatory elements and has previously been associated with significantly reduced promoter activity (58% promoter activation relative to wild-type). No pathogenic coding mutations or deletions were identified.

**Conclusion:**

Our findings suggest that this haplotype likely underlies the GH deficiency observed in the affected siblings. This represents the first report linking a homozygous *GH1* promoter haplotype to IGHD, underscoring the role of noncoding variants in endocrine disease.

## Introduction

Growth Hormone (GH) plays a fundamental role in human development, growth, and metabolism (1, 2). Its production and secretion by the anterior pituitary gland are highly regulated and complex processes; therefore, any disruption can result in GH deficiency (3). In children, congenital GH deficiency typically presents as decelerated linear growth and is frequently classified as idiopathic (2–4). However, some patients have a genetic cause for their disorder, due to mutations in one of several genes known to cause isolated (IGHD) or combined pituitary hormone deficiency (5, 6).

GH is encoded by the *GH1* gene, located on chromosome 17q23.3, within a cluster of five related genes (7). Its expression is regulated by upstream enhancer elements and a promoter region containing multiple transcription factor binding sites (8). The *GH1* regulatory and coding regions are highly polymorphic, with over 16 single nucleotide polymorphisms (SNPs) that can form haplotypes influencing transcriptional activity (9, 10). These polymorphisms span the promoter, coding, and noncoding regions, complicating the genetic architecture of *GH1* (11).

Mutations in the *GH1* coding sequence and whole gene deletions are well-established causes of inherited forms of IGHD, which typically occur without deficiencies of other pituitary hormones (5, 6). Despite extensive studies of *GH1* mutations, the cumulative effect of multiple promoter variants has not been linked to monogenic IGHD. Indeed, non-coding regulatory SNPs are often under-investigated, as their precise effect is difficult to determine, requiring extensive functional analysis, which is often not feasible in clinical routine (12). The aim of our study was to determine the genetic cause of IGHD in three siblings born to consanguineous parents.

## Material and methods

### Patients

We studied five individuals from a consanguineous Egyptian family, including three siblings diagnosed with IGHD and their unaffected parents, who were first cousins. The siblings presented normal motor and cognitive development but exhibited growth retardation and short stature. GH stimulation testing using clonidine and levodopa confirmed GH deficiency in the siblings (**Table 1**). Other pituitary hormone levels were normal, and brain magnetic resonance imaging (MRI) revealed no structural abnormalities. The two older siblings responded positively to recombinant GH therapy. Clinical data are summarized in **Table 1**.

**Table 1:** Clinical characteristics of the affected siblings.

Characteristics	Sibling 1	Sibling 2	Sibling 3
Identification number	#8477	#8478	#8479
Gender	Female	Male	Male
Current age	9.6 years	6.9 years	4.3 years
Age at diagnosis	5.5 years	3.5 years	2 years
Bone age (chronological/bone)	6/4 years	4/2.5 years	4/2.5 years
GH peak after stimulation (test)	0.19 µg/L (levodopa) 0.07 µg/L (clonidine)	0.14 µg/L (levodopa) 0.29 µg/L (clonidine)	0.77 µg/L (clonidine)
Height (Z-score) before treatment	- 4.2	- 6.4	- 3.1
Age at start of GH treatment	6 years	4 years	Not started yet
Initial GH dose	0.027 mg/kg/day	0.027 → 0.036 mg/kg/day	
Height (Z-score) after treatment	0.11 (after 43 months)	- 1.4 (after 34 months)	–
Brain and pituitary MRI	Normal	Normal	n/a
Other clinical problems	None	None	None

Parental heights: father 177.5 cm (58^th^ percentile), mother 159.0 cm (26^th^ percentile). Mid-parental height: male (siblings 2 and 3) 174.75 cm, female (sibling 1) 161.75 cm. Target height range (± 8.5 cm): male 166.25-183.25 cm, female 153.25-170.25 cm. GH, growth hormone; MRI, magnetic resonance imaging; n/a, not available.

The study was conducted in accordance with the Declaration of Helsinki and approved by the Ethics Committee of the Faculty of Health Sciences, University of Beira Interior (Ref: CE-FCS-2012-012). Informed consent was obtained from all subjects involved in this research.

### Genetic analysis

Genomic deoxyribonucleic acid (DNA) was extracted from peripheral blood leucocytes using previously described methods (13) and used with polymerase chain reaction (PCR) primers to amplify the *GH1* promoter and coding regions (NM_000515.5) (primer sequences and PCR conditions available upon request). Bidirectional sequencing of the PCR products was performed using a semi-automated capillary DNA sequencer (STAB VIDA, Caparica, Portugal; ABI 3730XL, Applied Biosystems; Thermo Fisher Scientific, Waltham, MA, USA). In order to exclude other genetic causes of GH deficiency, we applied additional steps of multiplex ligation-dependent probe amplification (MLPA) and whole-exome sequencing (WES). Specifically, in sibling 2, we analysed copy number variations, through an MLPA assay, using the SALSA MLPA probemix P216-C1 (MRC Holland, Amsterdam, Netherlands), according to the manufacturer’s guidelines. This kit includes probes for *GH1*, as well as for other GH deficiency-related genes (*POU1F1*, *PROP1*, *GHRHR*, *LHX3*, *LHX4*, and *HESX1*). Fragment analysis was performed with the DNA sequencer described above. In addition, WES was carried out on siblings 1 and 2, as previously described (14). Genetic sequence variants were filtered according to the following cumulative criteria: i) found in both siblings; ii) non-synonymous or located within ten intronic nucleotides adjacent to coding exons; iii) absent or rare (maximum allele frequency < 0.01) in the Genome Aggregation Database (v.4.1.0) (15); and iv) located in genes associated with GH deficiency in the Online Mendelian Inheritance in Man database (16). Variants were classified according to American College of Medical Genetics and Genomics/Association for Molecular Pathology criteria guidelines (17).

## Results

**Figure 1:**
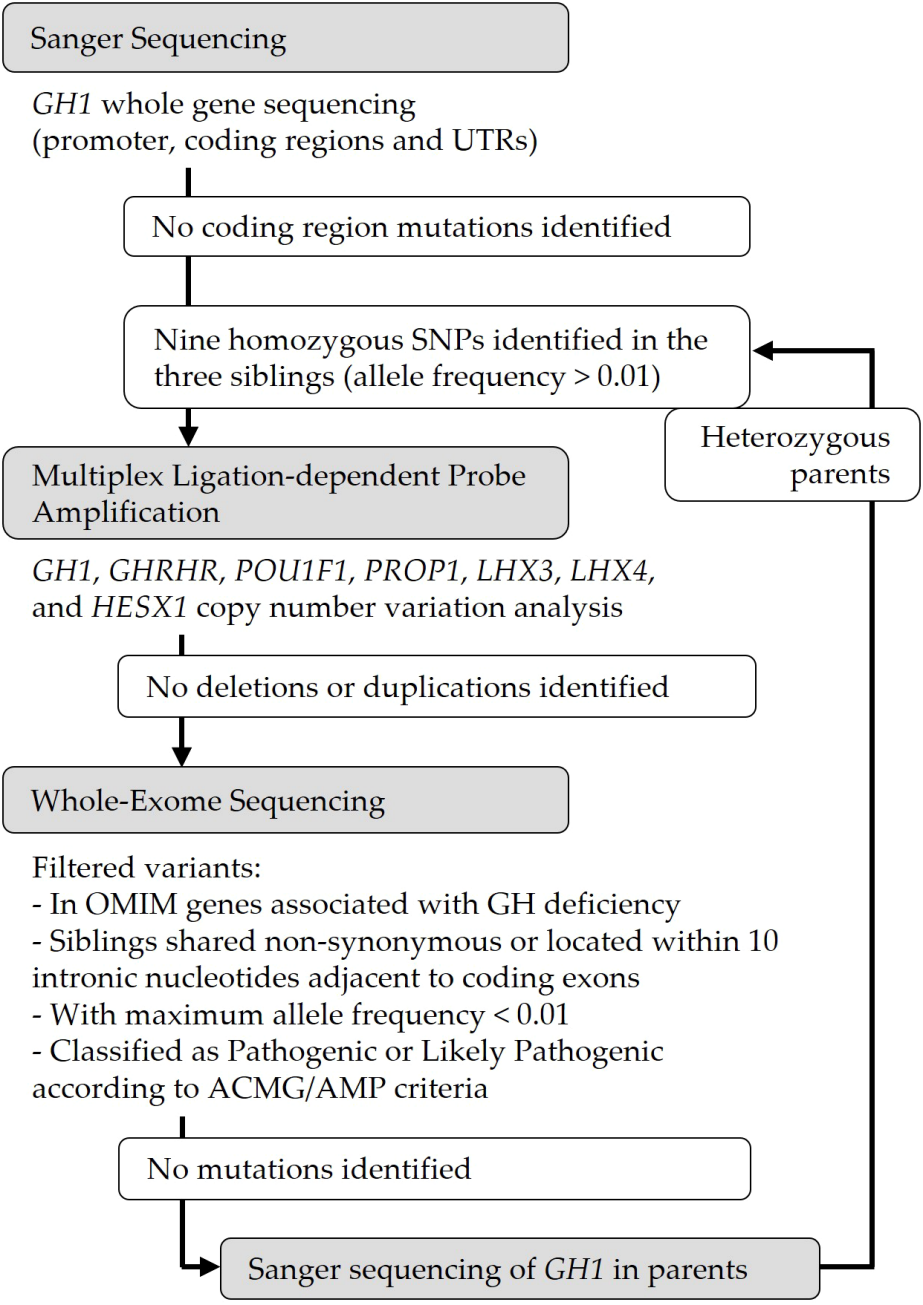
Flowchart of the genetic testing of a family with isolated growth hormone deficiency (IGHD). To identify the genetic cause in the siblings, targeted *GH1* sequencing, multiplex ligation-dependent probe amplification (MLPA) and whole-exome sequencing (WES) was performed. No pathogenic coding mutations, duplications or deletions were identified. The three siblings were homozygous for a shared set of polymorphisms across the *GH1* locus, and both parents were heterozygous for this haplotype. UTRs, untranslated regions; OMIM, Online Mendelian Inheritance in Man; GH, Growth Hormone; ACMG/AMP, American College of Medical Genetics and Genomics and Association for Molecular Pathology.

**Figure 2:**
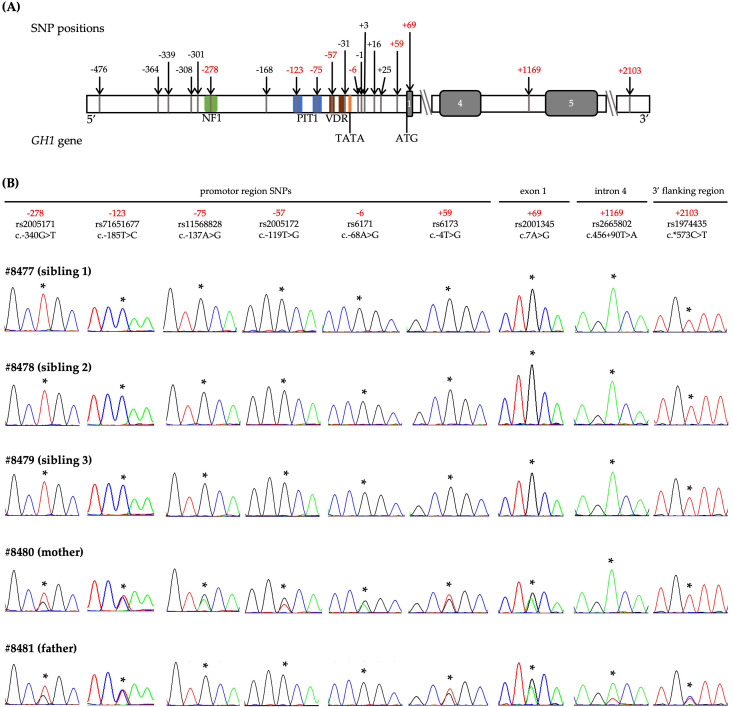
Genetic analysis of *GH1* in a family with isolated growth hormone deficiency. **(A)** Schematic representation of the *GH1* gene showing the positions of 20 known single nucleotide polymorphisms (SNPs) from –476 to +2103, numbered relative to the transcriptional start site, as described by Horan et al., 2003 (10). SNPs identified in the three affected siblings are highlighted in red. Exons are depicted as grey boxes. Coloured boxes indicate transcription factor binding sites: nuclear factor 1(NF1, in green), pituitary transcription factor 1 (PIT1, in blue), vitamin D receptor (VDR, in brown), and the TATA box (orange). **(B)** Sequence chromatograms of the nine SNP positions for all five family members. Asterisks (*) indicate the position of each SNP. The three affected siblings were homozygous for the rare haplotype, while both parents were heterozygous, consistent with autosomal recessive inheritance.

Based on the suspicion of autosomal recessive inheritance due to the parental consanguinity, we initially analysed the *GH1* gene in the three siblings. No pathogenic mutations were identified in the coding regions or canonical splice sites (**Figure 1**). However, all three siblings were homozygous for a shared set of nine previously reported SNPs across the *GH1* locus: −278T, −123C, −75G, −57G, −6G, +59G, +69G, +1169A, and +2103T, as numbered according to Horan et al. (10) (**Figure 2**). The population frequency of this haplotype has been estimated to be <1% (10). Both parents were heterozygous for this haplotype. Additional analyses, including MLPA and WES, did not reveal any other potentially pathogenic variants associated with GH deficiency (**Figure 1**).

## Discussion

Previous studies have demonstrated that polymorphisms in the *GH1* promoter can modulate gene expression and influence circulating GH levels (8, 10, 11, 18–23). To date, no homozygous haplotype has been clearly implicated in monogenic IGHD. In this study, we report three siblings with IGHD who share a homozygous *GH1* haplotype consisting of nine SNPs, distributed from the promoter to the 3′ flanking region.

Five of the SNPs (−278T, −75G, −57G, −6G, and +59G) comprise the promoter haplotype 21 described by Horan et al. (10). These authors identified 36 different *GH1* haplotypes from 15 SNPs (10, 24, 25) in a control population. Haplotype 21 had a population frequency of <1% and demonstrated only 58% promoter activation relative to wild-type in functional assays (10). These SNPs are located in regions essential for *GH1* transcriptional regulation (22). The -278T is located within the nuclear factor 1 (NF1) binding site, the -75G in the proximal pituitary transcription factor 1 (PIT1) binding site, the -57G in the vitamin D receptor (VDR) response element region, and the -6G at the transcriptional start site (22). Giordano et al. (19) specifically studied the -75G polymorphism and found reduced *GH1* transcription due to decreased PIT1 binding affinity. However, GH stimulation studies in individuals carrying this variant did not confirm a functional effect, likely due to insufficient homozygous sample size (19). These findings support the hypothesis that cumulative effects of multiple regulatory SNPs may reduce *GH1* transcription. Indeed, the impaired binding of essential transcription factors, caused by these SNPs, can weaken the promoter’s ability to recruit the necessary machinery for transcription (22).

Our patients also carried four additional SNPs (-123C, +69G, +1169A, and +2103T). The −123C SNP, located in a conserved Specificity Protein 1 (SP1) and PIT1 binding site, has been shown to have no significant impact on transcription in luciferase reporter assays (18, 23). The +69G SNP (p.Thr3Ala) is a missense variant located in the GH signal peptide. Though initially reported in GH-deficient patients (26), it is now considered a benign polymorphism (11, 18, 26, 27). Importantly, the intronic +1169A SNP, in cis with −278T and −57G, has been associated with reduced GH secretion and lower insulin-like growth factor 1 (IGF1) levels (28). Functional studies have demonstrated impaired *GH1* expression and GH secretion linked to this variant, although the effect may be influenced by adjacent regulatory SNPs (29). The +2103T SNP, located in the 3′ flanking region, has also been implicated in reduced GH secretion when present with −278T, −57G, and +1169A (29). Notably, Yamamoto et al. (30) identified this four-SNP combination in GH-deficient siblings, although their homozygous mother had normal stature, suggesting these variants alone may be insufficient to cause disease.

In our study, the homozygous co-occurrence of all nine variants — many with individually demonstrated or suspected functional impact — strongly suggests a cumulative regulatory effect on *GH1* expression. This may explain the phenotype observed in our patients and supports a role for the genetic screening of *GH1* promoter haplotypes in cases of IGHD lacking coding mutations.

Limitations of our study include the absence of *in vivo* functional data confirming reduced GH secretion associated with this haplotype. Such functional assays would be useful to validate our findings. While MLPA and WES excluded known GH deficiency-associated variants, we cannot rule out the involvement of unknown regulatory elements or genes. Nonetheless, the identification of a shared homozygous rare haplotype in three affected siblings provides compelling evidence for its pathogenicity.

In conclusion, we identified a rare homozygous *GH1* haplotype comprising nine regulatory and coding SNPs that may collectively impair *GH1* expression, representing the likely cause of IGHD in three siblings from a consanguineous family.

## Data Availability

The original contributions presented in the study are included in the article/supplementary material. Further inquiries can be directed to the corresponding author.
